# Intracranial calcification and psychotic symptoms after irradiation in a patient with Fanconi anemia: A case report

**DOI:** 10.1002/pcn5.10

**Published:** 2022-04-27

**Authors:** Soichiro Iki, Hitomi Shimizu, Yoshiro Morimoto, Naoki Yamamoto, Aiko Ohashi, Tatsuyuki Tayama, Akira Imamura, Hiroki Ozawa

**Affiliations:** ^1^ Department of Neuropsychiatry, Unit of Translational Medicine Nagasaki University Graduate School of Biomedical Sciences Nagasaki Japan; ^2^ Child and Adolescent Psychiatry Community Partnership Unit Nagasaki University Hospital Nagasaki Japan; ^3^ Department of Pediatrics Saiseikai Nagasaki Hospital Nagasaki Japan; ^4^ Department of Psychiatric Rehabilitation Science Nagasaki University Graduate School of Biomedical Sciences Nagasaki Japan

**Keywords:** cerebroretinal microangiopathy with calcifications and cysts (CRMCC), Fanconi anemia, idiopathic basal ganglia calcification, intracranial calcification, psychosis

## Abstract

**Background:**

Patients with Fanconi anemia (FA) are at high risk for the development of malignancies, and are often treated with radiation therapy. Radiation therapy during childhood can cause intracranial calcification after a latent period, which has been associated with psychiatric symptoms. Despite the high sensitivity of patients with FA to radiation, intracranial calcification has rarely been reported in these patients.

**Case Presentation:**

A 17‐year‐old girl presented with psychiatric symptoms and cognitive impairment. She had been diagnosed with FA at 3 years old, and had received a bone marrow transplant at 5 years old with a preparative regimen that included total body irradiation. Results of IQ tests revealed a characteristic pattern of decline between the ages of 15 and 17 years. Computed tomography indicated multiple intracranial calcifications in regions associated with psychotic symptoms, including the frontal lobe and thalamus. The patient's psychiatric symptoms improved with the administration of blonanserin.

**Limitations:**

The patient did not have regular intracranial imaging, making it difficult to confirm a direct relationship between intracranial calcification, psychiatric symptoms, and cognitive impairment. It is unclear whether the intracranial calcification in this case can be explained entirely by irradiation.

**Conclusion:**

This case suggests a link between FA, intracranial calcification, and psychosis, in which intracranial calcification may have caused psychiatric symptoms. At present, evidence regarding the characteristics of psychiatric symptoms of intracranial calcification and its treatment is lacking. The current case will be helpful for elucidating the pathogenesis of this disorder and developing appropriate treatment protocols.

## Graphical abstract

This case suggests a link between Fanconi anemia, intracranial calcifications, and psychosis, in which radiation‐induced intracranial calcifications may have caused the psychiatric symptoms.

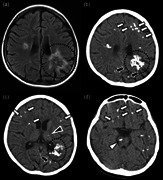

## BACKGROUND

Fanconi anemia (FA) is a congenital disorder characterized by progressive pancytopenia, transformation to myelodysplastic syndrome and acute myeloid leukemia, congenital morphological abnormalities, and susceptibility to carcinogenesis associated with impaired DNA damage repair.^1^ FA has a high incidence of malignancy, and patients often undergo radiation as a pretreatment for tumor therapy or treatment. However, FA patients are prone to serious adverse radiation events caused by impaired DNA‐damage response that makes them susceptible to malignancies and also increases their sensitivity to radiation.^2^, ^3^ In children, intracranial calcification occurs after a latent period as an adverse event of radiation therapy to the central nervous system.^4^ However, there have been no reports of intracranial calcification as a result of total body irradiation as a pretreatment for bone marrow transplantation for FA. Among the very diverse symptoms caused by FA, mental retardation is one of the most common neuropsychiatric symptoms. However, to the best of our knowledge, schizophrenia‐like psychiatric symptoms, such as hallucinations and delusions, have not previously been reported in FA patients.

Here, we report on a 17‐year‐old girl who was diagnosed with FA and treated with bone marrow transplantation with total body irradiation, including the brain, who developed psychotic symptoms during the course of the disease, and exhibited brain calcification in brain imaging results.

## CASE PRESENTATION

**Figure 1:**
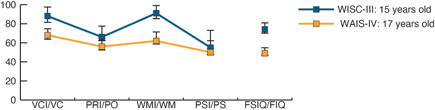
Summary of IQ test results. Although direct comparisons are difficult because of age‐related changes in test scales (WISC‐III at age 15 years, WAIS‐IV at age 17 years), the results suggested that the intellectual abilities of this patient may have declined at age 17 compared with age 15, particularly verbal comprehension and working memory. FIQ and FSIQ, full‐scale IQ; PO, perceptual organization; PRI, perceptual reasoning index; PS, processing speed; PSI, processing speed index; VC, verbal comprehension; VCI, verbal comprehension index; WM, working memory; WMI, working memory index.

**Figure 2:**
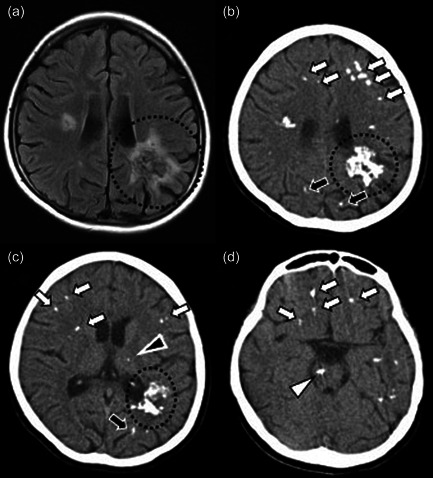
The patient's neuroradiographic findings. (a) Magnetic resonance axial fluid‐attenuated inversion recovery (FLAIR)‐weighted image of the body level of the lateral ventricle shows massive calcification in the white matter of the left parietal lobe (the area enclosed by the dashed circle) and part of the occipital lobe, with surrounding FLAIR high‐signal areas. (b) Computed tomography (CT) images of the same level showed multiple calcified lesions in addition to those identified in MRI. The calcified lesions in the frontal lobe (white arrow) and occipital lobe (black arrow) were scattered in dots. Axial CT images (c) at the level of the basal ganglia and (d) at the level of the lower midbrain also showed multiple calcified lesions. In addition to subcortical white matter and deep white matter, calcification was seen in the left thalamus (black arrowhead) and brain stem (white arrowhead).

The patient was born to a nonconsanguineous, healthy, young Japanese couple at 38 weeks and 4 days of gestational age with a birth weight of 2060 g (−2.36 standard deviations). At birth, absence of the radius and esophageal atresia were detected, and required surgery. Because thrombocytopenia was observed in the neonatal period and persisted until the age of 3 years, a bone marrow examination was performed, which resulted in the diagnosis of myelodysplastic syndrome. Accordingly, a chromosomal breakage test confirmed chromosomal instability, leading to the diagnosis of FA. At 5 years old, the patient received an unrelated bone marrow transplant with a preparative regimen of fludarabine, antithymocyte globulin, cyclophosphamide, and total body irradiation (4.5 Gy), which resulted in hematological remission. Brain magnetic resonance imaging (MRI) performed at this point showed no abnormal findings in the patient's central nervous system. Her younger sister was also diagnosed with FA. At the age of 8 years, the patient was diagnosed with left‐sided conductive hearing loss. No specific developmental abnormality was detected in infant medical checkups, and she was enrolled in regular classes in elementary and junior high school. At the age of 15 years, the patient came to our hospital for a developmental evaluation because her pediatrician suspected a delay in her mental development. The patient's full‐scale IQ as assessed by the Wechsler Intelligence Scale for Children 3rd edition (WISC‐III) was 74 (WISC‐III: full‐scale IQ = 74, 90% confidence interval [CI] = 71–81; verbal comprehension = 88, 90% CI = 82–98; perceptual organization = 66, 90% CI = 62–77; working memory = 91, 90% CI = 85–99; processing speed = 55, 90% CI = 54–73; Figure 1). At the age of 16 years, the patient underwent ophthalmic surgery for decreased vision caused by retinal neovascularization, ocular hypertension, and vitreous hemorrhage in both eyes. Up to this point, the patient had many friends and no episodes that had caused problems in her school life. She lived in the school dormitory and did not experience any difficulties. At 17 years of age, the patient was no longer able to hold a conversation and began to talk to herself more. Auditory hallucinations prompted her to take a bath with her clothes on. Because of these ongoing strange behaviors, she was referred to us by the pediatrician and was admitted to the hospital with her consent. There was no history of illegal drug or alcohol use. After admission, brain MRI was performed, which suggested multiple intracranial calcifications. Subsequently, a computed tomography (CT) scan showed calcifications in extensive areas of the brain, including the frontal lobe, parietal lobe, occipital lobe, basal ganglia, thalamus, midbrain, and brainstem (Figure 2). Electroencephalography revealed no abnormal signals indicating epilepsy or impaired consciousness.

The results of the Wechsler Adult Intelligence Scale 4th edition (WAIS‐IV) suggested a possible decline in intellectual ability compared with that at 15 years of age, although direct comparisons are difficult because of changes in the test scales associated with aging. Importantly, the results suggested a significant decline in verbal comprehension and working memory abilities (WIAIS‐IV: full‐scale IQ = 49, 90% CI = 46–55; Verbal Comprehension Index = 68, 90% CI = 64–75; Perceptual Reasoning Index = 56, 90% CI = 53–65; Working Memory Index = 62, 90% CI = 59–71; Processing Speed Index = 50, 90% CI = 48–62; Figure 1). Other neuropsychological tests showed deficits in a wide range of cognitive impairments, including frontal lobe function, visuospatial executive function, memory, attention, conceptual thinking, delayed replay, and disorientation (Frontal Assessment Battery: total = 7, conceptualization = 2, mental flexibility = 2, programming = 0, sensitivity to interference = 0, inhibitory control = 0, environmental autonomy = 3; Japanese version of the Montreal Cognitive Assessment: total = 8, visuospatial = 1, naming = 0, attention = 3, language = 0, abstraction = 1, delayed recall = 0, orientation = 3).

During the patient's hospitalization, she had interactive auditory hallucinations that seemed to advise her, and her behavior was influenced by these auditory hallucinations. She also complained of visual hallucinations of black shadows and ghosts. In addition, the patient had various psychiatric symptoms, such as delusions of reference, delusions of grandeur that she was the emperor's daughter, thought broadcasting, thought hearing, thought withdrawal, and delusional perception. These psychiatric symptoms did not change with the change in living environment associated with hospitalization. The patient's symptoms, especially the various cognitive impairments, were consistent with symptoms caused by intracranial calcification (e.g., frontal lobe, left parietal lobe, and occipital lobe) identified by brain imaging, and there were no other obvious triggers, such as significant stress or drug abuse. Although the patient originally had a relatively low IQ, her characteristic cognitive decline and stress‐ and environment‐independent psychiatric symptoms that had recently emerged did not appear to be symptoms of her mental retardation. Thus, her psychiatric symptoms were diagnosed as an organic delusional disorder based on *International Statistical Classification of Diseases and Related Health Problems*, 10th Revision. Schizophrenia was considered as a possible differential disease, but was not diagnosed because of organic factors strongly suspected to be associated with her symptoms. In addition, rare genetic diseases, such as chromosomal instability syndromes and cerebroretinal microangiopathy with calcifications and cysts (CRMCC) were also considered to be important for differential diagnosis, so genetic testing was considered necessary. However, although a genetic counselor explained the importance of the genetic testing, the patient and family chose not to give their consent because of various concerns about genomic analysis (e.g., possible discrimination, psychological stress of testing).

The patient's Positive and Negative Syndrome Scale (PANSS) score was 48 on admission and increased to 55 without improvement with olanzapine. After switching from olanzapine to blonanserin, the patient's symptoms improved and her PANSS score decreased to 36, meaning that she could be managed as an outpatient. With the administration of olanzapine and blonanserin, the patient exhibited very slight extrapyramidal symptoms (EPS), with almost no problems in her daily life (Drug‐Induced Extrapyramidal Symptoms Scale: before administration, 0; after administration, 2–3; Table 1).

**Table 1:** Summary of the patient's medications and psychopathological and extrapyramidal symptoms

	Medications			
	Olanzapine (3 mg/day)	Olanzapine (20 mg/day)	Olanzapine (20 mg/day) ＋ Blonanserin (8 mg/day)	Olanzapine (10 mg/day) ＋ Blonanserin (12 mg/day)
Hospital day	1st	35th	42nd	56th
DIEPSS	0	2	3	3
Positive scale	10	17	12	4
Negative scale	14	10	9	8
General psychopathology scale	24	28	27	24
PANSS	48	55	48	36

Abbreviations: DIEPSS, Drug‐Induced Extrapyramidal Symptoms Scale; PANSS, Positive and Negative Syndrome Scale.

## DISCUSSION

Here, we have reported a case of intracranial calcification in a patient with FA, and cognitive dysfunction and schizophrenia‐like symptoms that may have been caused by calcified lesions. To the best of our knowledge, this is the first reported case of psychiatric symptoms in a patient with FA with multiple intracranial calcifications. Although a high percentage of patients with FA (65%–90%) have been reported to exhibit pathological brain imaging findings, intracranial calcification is rare (0.0%–9.0%) and, when observed, is typically mild.^5^, ^6^ However, considering that intracranial calcification has been reported to occur as a late effect of radiotherapy of the central nervous system in children,^4^ and that FA patients are highly sensitive to radiation, it is possible that this patient presented with intracranial calcification as a late side‐effect of the total body irradiation she received as a pretreatment for bone marrow transplantation.

However, this case involved several serious limitations that should be considered. The patient had not undergone routine intracranial imaging, and the lack of detailed data regarding the timing of intracranial calcification occurrence makes it difficult to confirm a direct relationship between intracranial calcification and her various symptoms, especially psychiatric symptoms, such as auditory hallucinations. Considering the high incidence of intracranial complications in FA and the usefulness of intracranial abnormality detection in the evaluation of the endocrine system,^5^, ^6^ this case highlights that regular intracranial imaging is beneficial for FA patients. The difficulty confirming a causal relationship between intracranial calcification and irradiation is another limitation of the current study. The type of intracranial calcification seen in postirradiation patients is primarily located in the basal ganglia, whereas multiple calcifications, such as those in the current case, are not usually seen.^7^ In addition, to the best of our knowledge, there is little evidence that total body irradiation as a preparative regimen for bone marrow transplantation in children causes multiple intracranial calcifications.^6^ Thus, it remains unclear whether all of the calcifications in this case were radiation‐induced. When other symptoms, such as ocular symptoms, are included, it is necessary to consider a pathogenesis that cannot be explained solely by the chromosomal instability associated with FA.

CRMCC (OMIM #612199, #617341) is caused by telomere maintenance dysfunction, and the phenotype is very similar to FA, with intracranial calcification that is very similar to that observed in the current case. The chromosomal instability observed in FA is known to be related to telomere maintenance mechanisms. For example, the protein encoded by *FANCD2*, the causative gene of FA, in the complementation group D2 (OMIM # 227646), has been shown to co‐localize with the unique telomere protein TRF1. Depletion of FANCA and FANCD2 results in telomere loss and reduced telomere sister chromatid exchange. In addition, FANCC, the protein encoded by the gene responsible for FA in complementation group C (OMIM # 227645), is thought to be involved in the maintenance of telomere length during homologous recombination mediated an alternate lengthening of telomeres.^8^ Accordingly, some types of FA are associated with abnormal telomere maintenance function. Telomere length measurements by flow‐fluorescence in situ hybridization or Southern blot and genetic testing are very useful for differentiating FA from clinically similar diseases, such as CRMCC, and such genetic testing may have been beneficial in elucidating the mechanisms of our patient's atypical intracranial calcification and psychiatric/cognitive symptoms. However, as declared in the Lisbon Declaration on the Rights of Patients,^9^ patients have the right to refuse genomic testing. Although the genetic approach would have been useful in the present case, we were not able to perform this analysis to confirm the diagnosis.

There have been several reports of psychiatric symptoms associated with intracranial calcification, particularly in the early‐onset form of idiopathic basal ganglia calcification (IBGC), which is classically known as Fahr's syndrome.^10^ In as many as 40% of IBGC patients, the initial manifestation of the disorder is psychiatric symptoms, including auditory hallucinations, complex visual hallucinations, and paranoid delusions.^11^, ^12^ It has been hypothesized that positive symptoms of schizophrenia, such as hallucinations and delusions, are the result of increased activity of the subcortical dopamine system caused by dysfunction of the larger hippocampal–midbrain–striatal circuit. Many regions are involved in maintaining this large circuit, including the medial prefrontal cortex, ventral pallidum, thalamic nuclei, and medial septum.^13^ In the present case, the subcortical white matter, including the frontal lobe, and the thalamus contained multiple calcifications, suggesting that there was some disruption in the circuitry associated with the positive symptoms of schizophrenia. In addition, previous reports of reduced structural connectivity in the left parietal lobe in patients with schizophrenia and psychotic bipolar disorder^14^ suggest that a large calcified lesion in the left parietal lobe may have been associated with psychiatric symptoms in this case. In patients with IBGC and psychosis, antipsychotics can easily cause EPS, and psychosis responds poorly to medication.^15^ Because our patient was also considered to be at high risk for EPS, we used second‐generation antipsychotics that are effective for positive symptoms and less likely to cause EPS. Initially, we chose olanzapine, a multi‐acting receptor‐targeted antipsychotic, but it was ineffective. Blonanserin, a serotonin–dopamine antagonist, was effective. Clozapine is another candidate antipsychotic that is less likely to cause EPS. However, to the best of our knowledge, data regarding the efficacy and safety of clozapine for psychiatric symptoms in patients with IBGC^16^ or FA are very limited. In addition, considering a previous report of a high rate of anemia in patients taking clozapine,^17^ we believe that the use of clozapine in FA patients should be considered with caution. At present, there is very little evidence regarding the choice of antipsychotic drugs for the psychiatric symptoms derived from intracranial calcification, and further data are needed to determine treatments that are safe and effective.

## CONCLUSION

In summary, this case report suggests that intracranial calcification after total body irradiation in an FA patient may have caused psychiatric symptoms. At present, evidence regarding the characteristics of psychiatric symptoms of intracranial calcification and its treatment is lacking, and data from more cases are needed to develop appropriate treatment protocols. FA is a disease with diverse and severe symptoms, and involves a major impact on the lives of patients and their families. We hope that the current case study will contribute to the development of a comprehensive care strategy to improve the quality of life of patients with FA.

## AUTHOR CONTRIBUTIONS

Soichiro Iki treated the patient and drafted the manuscript. Hitomi Shimizu and Yoshiro Morimoto critically reviewed the draft and revised it. All authors made substantial contributions, drafted the manuscript, and approved the final manuscript.

## CONFLICTS OF INTEREST

The authors declare no conflicts of interest.

## ETHICS APPROVAL STATEMENT

Informed consent was obtained from the participant. We have obtained a release from the participant and the family giving us permission to publish.

## Data Availability

Data sharing is not applicable to this article as no datasets were generated or analyzed for the present study.
